# Fabrication of Functional Microdevices in SU-8 by Multi-Photon Lithography

**DOI:** 10.3390/mi12050472

**Published:** 2021-04-21

**Authors:** Pooria Golvari, Stephen M. Kuebler

**Affiliations:** 1 Chemistry Department, University of Central Florida, Orlando, FL 32816, USA; pgolvari@knights.ucf.edu; 2 CREOL, The College of Optics and Photonics, University of Central Florida, Orlando, FL 32816, USA; 3 Department of Material Science and Engineering, University of Central Florida, Orlando, FL 32816, USA

**Keywords:** multi-photon lithography, SU-8, micro-fluidics, micro-robotics, MEMS, metallization, direct laser writing

## Abstract

This review surveys advances in the fabrication of functional microdevices by multi-photon lithography (MPL) using the SU-8 material system. Microdevices created by MPL in SU-8 have been key to progress in the fields of micro-fluidics, micro-electromechanical systems (MEMS), micro-robotics, and photonics. The review discusses components, properties, and processing of SU-8 within the context of MPL. Emphasis is focused on advances within the last five years, but the discussion also includes relevant developments outside this period in MPL and the processing of SU-8. Novel methods for improving resolution of MPL using SU-8 and discussed, along with methods for functionalizing structures after fabrication.

## 1. Multi-Photon Lithography

MPL is an increasingly versatile technique for fabricating 3D micro- and nanostructures based on multi-photon absorption (MPA) activated with a tightly focused pulsed laser. MPL has been performed with a wide range of materials, including photopolymers, chalcogenide glasses, as well as ceramic/polymer and metal/polymer composites [1,2,3,4]. MPL using SU-8 has enabled significant advances in micro-device fabrication, due to the novel properties of SU-8 and its ability to be activated by MPA. The non-linear optical response of the photoresist strongly confines photochemical activation within the 3D volume of the material to a region centered around the focal spot. Non-linear chemical response of the material to the local irradiance profile confines the material transformation and enables high-resolution 3D patterning.

### 1.1. Multi-Photon Absorption

The simultaneous absorption of photons having the sum energy required for a transition was predicted by Maria Göppert-Mayer as early as 1930 [5,6]. Her prediction was experimentally verified three decades later [7] with the invention of optical masers. Two-photon absorption (2PA) is the simplest form of MPA, in which two photons promote a molecule to a higher electronic state, if their sum energy equals the transition energy [8]. The first photon excites the molecule to a very short-lived virtual state (femtosecond lifetime) whereupon a second photon can co-absorb nearly simultaneously [8,9]. The probability of 2PA correlates with the square of photon flux because it requires two photons to arrive at the molecule at virtually the same time. Ultrafast pulsed lasers with low duty-cycle can deliver femto-second, high-intensity bursts of photons to excite molecules locally and achieve MPA, without damaging the material.

The quadratic dependence on irradiance creates a subwavelength focal volume in which the rate of 2PA is high. This is in contrast with one-photon absorption (1PA), for which the excitation rate has a linear dependence on irradiance that simply follows the beam profile. A focused visible or near-infrared (NIR) beam can travel through a material containing UV-absorbing chromophores and photoinitiators without substantial 1PA. But near the focal point, where the irradiance is highest, the rate of 2PA can exceed that of 1PA generating a region of significant photoexcitation that is strongly localized in 3D around the focal spot.

The field of microscopy was first to benefit from 2PA when sub-picosecond pulses of a red-emitting dye laser were used to image sub-wavelength features [10]. Soon after, the same group reported fabrication of high aspect-ratio 2D micro-trenches based on 2PA [11]. A few years later, the Kawata group showcased the seminal work on 2PA-based 3D microfabrication [12] using femtosecond pulsed Ti:sapphire laser to create micro-features in a commercial urethane acrylic resin. This brought forth a new scheme for creating 3D micro-structures called multi-photon lithography (MPL).

### 1.2. Photopolymerization Lithography

Most photolithographic techniques rely on controllably changing the solubility of the photoresist upon exposure to define patterns. If the exposed regions become insoluble (i.e., negative photoresist) the final structure will replicate the exposure pattern. If exposure increases solubility, a pattern complementary to exposure will realize after development (i.e., positive photoresist). The contrast in solubility is usually driven by photo-activated chemistries such as photopolymerization. The kinetics of photopolymerization is well-known [13] and involves three main processes: initiation, propagation, and termination. The excited photoinitiators (PIs) undergo bond cleavage to create new species, through various possible mechanisms that depend on the specific molecule. For example, benzoyl-substituted radical initiators typically undergo intersystem crossing to the triplet state, followed by homolytic bond cleavage forming one or two active initiating free-radicals [13,14]. The active species can add on to monomers and grow a polymer, i.e., propagate. Eventually the active species are terminated due to recombination, oxygen quenching, and/or reaction with inhibitors within the resin.

### 1.3. Chemical Non-Linearity

In MPL the optical non-linearity in the PI/beam interaction is complemented by further chemical thresholding of the activated photochemistry. The basic idea is that significant material transformation occurs only when the local irradiance exceeds a certain threshold, that is material dependent. This effect is attributed to the quenchers in the resin (e.g., dissolved oxygen) which terminate photochemistry and can be replenished by diffusion if exposure time is long [15,16]. The PIs are excited in the focus of the laser beams where they can initiate polymerization. However, a minimum number of excited PI is required to create enough activated species to overcome the quenching barrier and initiate polymerization. As a result, regions where the PI is activated but sufficient polymerization does not occur will be washed away during development. Hence, solubility shows a threshold behavior with respect to the exposure dose. This so-called chemical non-linearity further decreases the dimensions of the polymerized feature.

### 1.4. Implementing MPL

**Figure 1:**
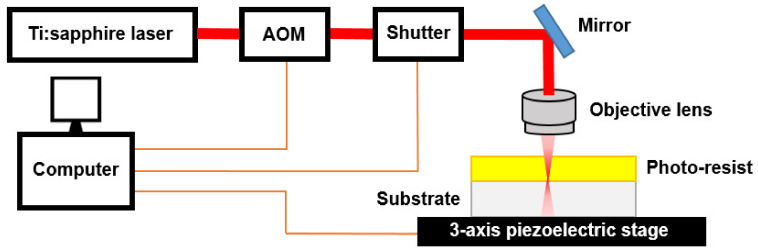
Schematic illustration of a typical MPL setup.

A typical set-up for MPL is shown in Figure 1. The output of a mode-locked femto-second laser is focused through a high-numerical-aperture (*NA*) objective lens to locally excite a PI and initiate photochemistry. An acousto-optic modulator (AOM) can be used to control the power that couples into the objective lens. By modulating the power, the relative size of the polymerized volume elements (voxels) can be controllably varied. A three-axis nano-positioner driven by a piezoelectric actuator is used to change the position of the sample with respect to the focal spot to define 3D patterns. Alternatively, the beam can be moved relative to the sample using galvanometer optical scanners [17,18,19].

In the literature, investigators commonly refer to the MPL technique as two-photon polymerization, two-photon lithography, multi-photon polymerization, and/or femtosecond direct laser writing. The name MPL is preferred because (a) higher-order nonlinear processes, and not just 2PA, commonly occur in the PIs and sensitizer used in MPL, and (b) while MPL is typically enabled by MPA-based photopolymerization, MPA-based micro-fabrication is also possible via other photochemistries such as 2PA-assisted reduction [3,20,21,22] or 2PA-assisted patterning of chalcogenide films [4,23,24,25]. Recent literature on MPL is immense with numerous groups around the world advancing the-state-of-the-art every year. Comprehensive reviews on MPL state-of-the-art have recently been published elsewhere [1,3,9,19,26,27,28,29,30].

## 2. SU-8 as a Material System for MPL

SU-8 is a negative-tone epoxy-based photoresist originally developed by IBM [31,32] and sold by Shell chemicals. SU-8 has become widely applied in fabrication of high aspect-ratio structures via photolithography with applications in micro-electromechanical systems (MEMS) and microfluidic devices, due to its chemical inertness, mechanical robustness, and high transparency. Soon after its commercialization, SU-8 3D structures were fabricated using MPL [33]. Since then, SU-8 has been frequently used as a popular resist for MPL of 3D functional device.

SU-8 can be spin-coated onto silicon, glass, ceramics, and metal with good adhesion to the surface. Nonetheless, dilute solutions of epoxy-functionalized silanes such as 3-glycidoxypropyl-trimethoxysilane (CAS# 2530-83-8) can be used to promote adhesion of polymerized SU-8 to glass substrates [34]. After spin-coating, the solvent is evaporated away with a pre-exposure bake to afford a dry resist layer. The SU-8 film is then patterned by MPL. A post-exposure baking (typically at 95 °C) is required to thermally activate the cationic polymerization. Unpolymerized SU-8 is then removed by immersion in a solvent, typically 1-methoxy-2-propanol acetate (CAS# 108-65-6), leaving behind the targeted structure.

### 2.1. Components of SU-8

**Figure 2:**
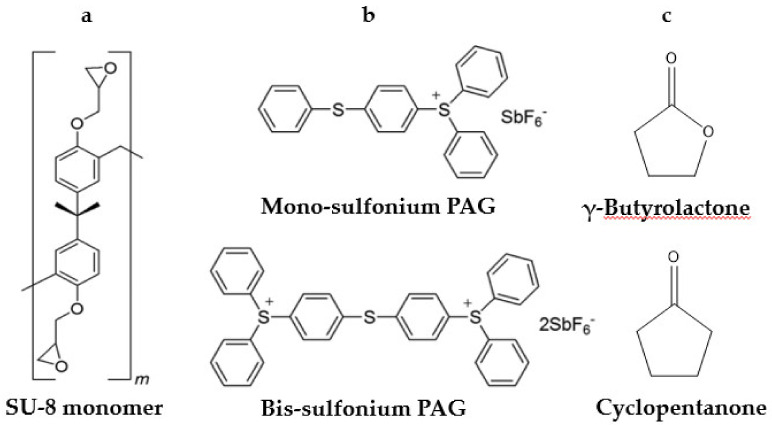
The components of SU-8: (**a**) SU-8 monomer; (**b**) sulfonium PAGs; (**c**) solvent. Adapted from Ref. [35] with the permission of AIP Publishing.

Currently, Kayaku Advanced Materials, Inc. (Westborough, MA, USA, formerly MicroChem) and Gersteltec Sàrl (Pully, Switzerland) are licensed suppliers of SU-8- resins. Various formulations are provided tailored to specific applications. The main components of SU-8 resins are similar and consist of: (1) epoxide-functionalized oligomers of bisphenol A; (2) a cationic photoinitiator based on sulfonium photoacid generators (PAGs); and (3) solvent added to enable spin-coating. The components are depicted in Figure 2. In some formulations, plasticizers like propylene carbonate are also added to improve processability.

#### 2.1.1. Oligomers

SU-8 consists of short oligomers of cross-linkable epoxides based on repeat units of bisphenol A diglycidyl ether (see Figure 2a). The epoxides undergo cationic ring-opening polymerization in the presence of Brønsted acids or other cations. The statistical average of epoxy groups per oligomer is eight, hence the name “SU-8.” In fact, commercial SU-8 contains shorter oligomers (i.e., SU-2, SU-4 and SU-8) as well as much heavier oligomers. Denning et al. reported that the molecular range of the species in SU-8 spans from 380 to 100,000 g mol^−1^ [36]. They also demonstrated that the lighter components are less likely to be incorporated into the cross-linked network and at lower exposures will be washed away during development. Heavy SU-8 (HSU-8) can be prepared by multiple extractions of smaller epoxides and mixed with a plasticizer for processability. HSU-8 demonstrates drastically lower mass-loss shrinkage, especially for exposures below the solubility threshold [36].

#### 2.1.2. Photoacid Generator

The photoinitiator for cationic polymerization of SU-8 is typically a photoacid generator (PAG). PAGs are light-sensitive molecules that form Brønsted acid following photoexcitation [37]. Common PAGs are based on aryl sulfonium (Figure 2b) and aryl iodonium salts. The effectiveness of a PAG in MPL is determined by three characteristics: MPA cross-section, quantum yield, and initiation efficiency [38]. Denoted by *σ_n_*, the MPA cross-section is a measure of the effective photon-capturing area of a molecule for a given *n*th-order absorption process, with units of m^2*n*^ s^(*n* − 1)^ molecule^−1^ photon^-(*n*−1)^. The TPA cross section *σ*_2_ is often represented as “*δ*” and has units of m^4^ s molecule^−1^ photon^−1^, typically expressed in units of “GM” (1 GM = 1 × 10^−58^ m^4^ s molecule^−1^ photon^−1^). Augmenting polarizability of PAGs by judicious incorporation of donor/accepter moieties can increase *δ* up to two orders of magnitude [28,38,39,40] compared to that of common UV initiators [41]. Increasing *δ* enhances the sensitivity of the resin and enables fabrication at lower powers. This is especially crucial in MPL of proteins and biological resins, where high powers can damage the sample [42,43,44]. Sensitizers like isopropylthioxanthone (ITX, CAS# 81-88-9) have been added to SU-8 to increase sensitivity toward MPA in the NIR region and lower the threshold power (*P*_th_) for polymerization [34,38,45]. ITX sensitizes SU-8 PAGs by electron transfer from its excited state. It can also be used as the Norrish type II sole initiator in acrylate polymerization showing highly non-linear absorption [15]. The quantum yield for acid-generation (*ϕ*_acid_) specifies the fraction of excited PAGs that form acid. The value of *ϕ*_acid_ impacts the efficacy of PAGs to initiate cationic polymerization. Aryl sulfonium PAGs used in SU-8 generally exhibit high yields (*ϕ*_acid_ > 0.5) [46]. The initiation efficiency *f* gives the fraction of generated species upon breakdown of the PI that can successfully initiate polymerization. Side reactions like recombination of initiator fragments or induced decomposition due to chain-transfer can cause inefficient initiation (*f* < 1) [13]. In PAGs, *f* is determined by the ability of the Brønsted acid to initiate cationic polymerization. The metal halide counter ion plays an important role in determining *f* of the PAG. In the case of SU-8, the counter ion hexafluoroantimonate stabilizes the acid so that it can initiate the epoxide. Nucleophilic counter ions are not desired because they lower *f* by attacking the epoxides. For a comprehensive discussion of PAGs the reader is referred to a recent review published elsewhere [47].

The PAG system used SU-8 is typically the mono-sulfonium and bis-sulfonium compounds 4-(phenylthio)phenyldiphenylsulfonium hexafluoroantimonate (CAS# 71449-78-0) and bis [4-(diphenylsulfonio)phenyl] sulfide bis(hexafluoroantimonate) (CAS# 89452-37-9) (see Figure 2b). The number of photons absorbed upon excitation, or the “order of excitation”, depends on the electronic structure of the PAG, as well as parameters of MPL that include excitation wavelength (*λ*), average power (*P*), and pulse-duration (*τ*_p_) of the laser beam. Williams et al. investigated the order of multi-photon excitation for sulfonium PAGs used in SU-8. The team measured the composition of SU-8 2075 and found that the PAGs consisted of 83 mol-% mono-sulfonium. A similar value of 89 mol-% was reported for commercial sources of the sulfonium PAGs alone [35]. This suggests that the photo-physics of SU-8 should be dominated by those of the mono-sulfonium PAG. Power dependent line-width studies were carried out at various writing speeds (*v*). By using the established relationship between the width of lines and average laser power *P*, Williams et al. obtained *P*_th_ for each value of *v*. The order of excitation is then extracted from the slope of a plot of ln[*P*_th_] versus *v*. Using this methodology, the order of excitation for the PAG in SU-8 2075 was found to be 2.89 ± 0.06 [48]. This *apparent* value suggests that three-photon absorption (3PA) dominates at *λ* = 800 and *τ*_p_ = 120 fs, accompanied by some 2PA. Z-scan measurements revealed that 2PA dominates for *λ* = 500–700 nm. Quantum chemical calculations supported these conclusions [35]. Importantly, it was shown that the order of excitation of SU-8 PAGs does not depend on the repetition rate of the laser (1 kHz from amplified femtosecond-pulsed system or 76 MHz from mode-locked laser), but it is strongly affected by pulse-width variation. MPL linewidth measurement using stretched (190 fs) yielded *n* = 3.8, suggesting dominance of 4PA [35]. Others have also reported 4PA using a similar setup with 180 fs pulses [49].

#### 2.1.3. Solvent

Solvent blending is used to control the viscosity of SU-8 monomer so that it can be easily processed onto a substrate, typically by spin-coating. Commercial vendors provide different formulation with various solvent contents. Increasing solvent content reduces viscosity and enables thinner layers of SU-8 to be spin-coated reproducibly. In early formulations like MicroChem’s SU-8 series, γ-butyrolactone was used as the solvent, with propylene carbonate added as plasticizer. In later formulations (Kayaku SU-8 2000 series) cyclopentanone was used to reduce drying times and improve adhesion to the surface. Figure 2c shows the structure of these two solvents. SU-8 2075 is a high-viscosity formulation commonly used in MPL which contains 19 wt.-% cyclopentanone. To characterize solvent content, chemical methods like proton nuclear magnetic resonance (^1^H NMR) spectroscopy [50] and Fourier transform infrared spectroscopy (FTIR) [51,52] are preferred to gravimetric methods [53,54], especially at low solvent contents, because they are not confounded by mass transfer/loss during handling.

### 2.2. Processing SU-8

Residual solvent remaining in the dry film affects its properties and behavior during exposure and post-exposure baking. High solvent content facilitates photoacid diffusion and lowers resolution [50,53]. Kuebler et al. showed that pre-exposure solvent content as low as 1 wt.-% causes features in woodpile structures to become rounded and less well resolved, whereas reducing solvent content down to 0.68 wt.-% yielded higher resolution structures with more sharply defined features [50,55]. While hot-plate heating is typically employed, alternative heating methods like IR and frequency variable microwave heating can also be used to reduce heating times and achieve lower solvent contents [56,57]. Convection oven heating is not recommended, because a skin of dry resin will form at the interface with air, which limits the vaporization of the solvent.

### 2.3. Properties of SU-8

**Figure 3:**
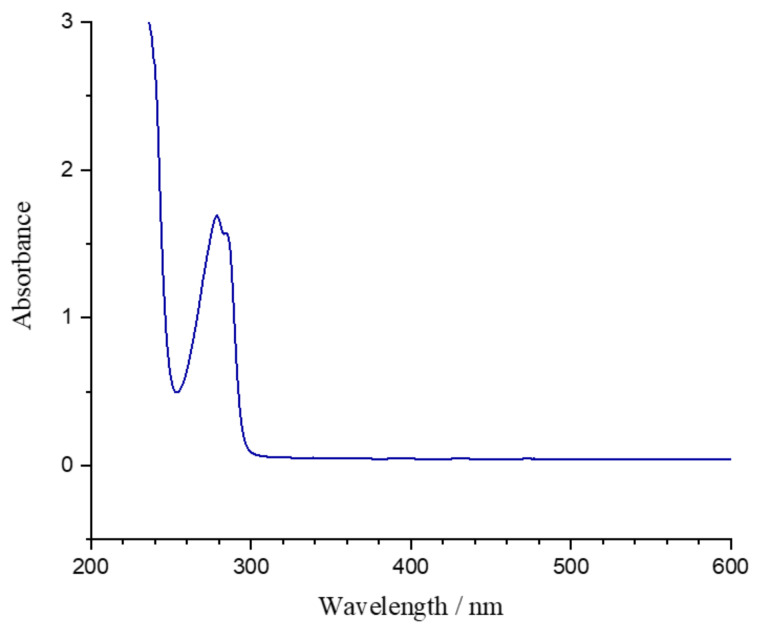
Optical absorption spectrum of SU-8 resin.

Figure 3 shows the absorption spectrum of SU-8 resin (as supplied by MicroChem). The material is largely transparent at visible wavelengths. The absorption band centered around 280 nm is the lowest-energy band of the mono-sulfonium PAG. Digaum et al. used ellipsometry to measure the complex refractive index of SU-8 polymerized films [58]. At 800 nm, the wavelength commonly used for MPL, the pre-baked and exposed SU-8 have refractive indices of 1.589 and 1.586, respectively [58,59]. Williams et al. showed that a difference in refractive index between SU-8 and the immersion oil used to match refractive index of optics in the objective lens introduces spherical aberration that increases the size of features with distance from the SU-8/immersion-oil interface. This effect is especially pronounced at powers close to *P*_th_, and can be partly corrected by power compensation [59].

The mechanical properties of polymerized SU-8 have been extensively studied [52,60,61,62]. Nano-indentation, micro-bending, and tensile analysis have been used to estimate the elastic modulus (*E*) of polymerized SU-8 [60]. *E* is complicated function of processing conditions (e.g., pre-baking), degree of cross-linking, and strain rate [61]. Typical values for *E* of SU-8 lie between 3 and 4 GPa [51,52] and *E* increases as strain rate decreases. *E* is also a function of moisture content of the polymerized SU-8 [51]. It was reported that post-exposure hard-baking (beyond glass transition temperature of SU-8 at 210 °C) increases *E* by reducing moisture content of the polymer [52]. Recently, Lemma et al. investigated bending stiffness of free-standing pillars fabricated by MPL using SU-8 and some acrylate resins designed specifically for MPL (IP series, Nanoscribe). The authors concluded that SU-8 pillars have larger *E* compared to acrylic pillars fabricated at the same power. However, resins from the IP series showed much larger dynamic range for MPL [63]. Advances in SU-8 based lithography have been reviewed elsewhere [28,64,65,66].

## 3. SU-8 Devices Fabricated by Multi-Photon Lithography

### 3.1. SU-8 Microfluidic Devices

**Figure 4:**
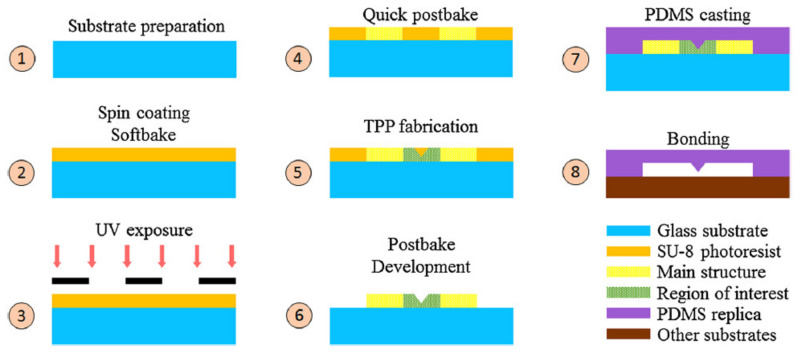
Illustration of a hybrid fabrication process based on photolithography and MPL. Reprinted from Ref. [71] by permission from Springer Nature: Springer-Verlag GmbH, Microfluidics and Nanofluidics Copyright 2018.

Microfluidics is the study and manipulation of liquids in small channels where at least one dimension is confined to a few microns [67,68,69]. Liquids behave differently in this size regime because surface phenomena overtake bulk behavior. Conventional photolithography and soft lithography are most commonly used to create micro-channels. For applications where higher resolution (few nm) is needed, more elaborate and expensive methods like electron beam lithography (EBL) are used. The marriage of MPL with photolithography enables fabrication of low-cost, high-resolution microfluidics. Normally, the microchannel platform is fabricated by mask photolithography (mm sized platforms with features of few tens of microns). Photolithography enables fast production of platforms using the same mask (typically fabricated by EBL). Then, complex 3D sub-micron features are added to the platform by MPL [70,71,72,73]. The final structure can be used as a replica to create micro- and nano- fluidic devices using soft lithography typically in polydimethylsiloxane (PDMS). A schematic process is shown in Figure 4.

**Figure 5:**
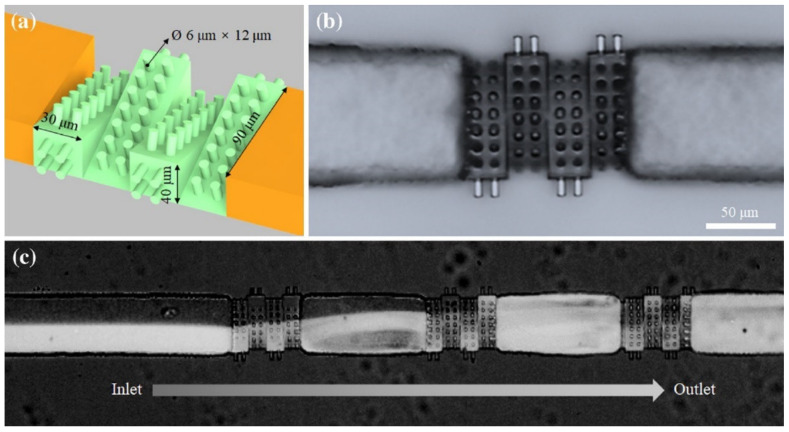
A passive mixer fabricated by MPL incorporated into a microfluidic platform. (**a**) Schematic illustration the four triangular blocks used as the mixer replica. (**b**) The replicated PDMS device. (**c**) Successful mixing of two liquids (fluorescein sodium salt and DI water) by using the passive mixer. Reprinted from Ref. [71] by permission from Springer Nature: Springer-Verlag GmbH, Microfluidics and Nanofluidics Copyright 2018.

Due to the small size of the micro-channels, laminar flow is dominant in the liquid and effective mixing does not occur. Passive mixers induce turbulent flow by merely varying the medium through which two or several liquids flow. Lin et al. reported a PDMS passive micro-mixer fabricated using an SU-8 replica [71]. Master SU-8 molds were fabricated using a hybrid scheme. Channels were first made by photolithography to reduce the fabrication time. Samples were then post-baked and immersed in oil, which has same refractive index as glass, to visualize the channels. Complex mixer structures (Figure 5) were then created by MPL. Finally, PDMS molds were used to replicate the mixers [71].

**Figure 6:**
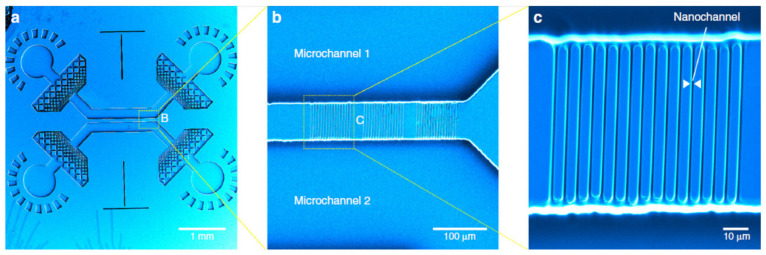
SEM of the nano-fluidic device fabricated by photolithography and MPL. (**a**) Device after PDMS molding. (**b**) 75 μm nano-channels connecting the two micro-channels. (**c**) Magnified view showing the 420-nm wide nano-channels. Figure reproduced from Ref. [72] under CC BY 4.0.

Oellers et al. used the hybrid procedure described above to fabricate an active microfluidic mixer. The mixer structure was fabricated by MPL such that it could swap the liquids entering from different inlets by directing their flow [74]. An analogous approach was adopted by Vanderpoorten et al. to fabricate an integrated nano-fluidic device. Master SU-8 micro-chambers were fabricated using mask photolithography. Using the refractive index change created after writing the pre-exposed areas, nano-channels (420-nm wide) were fabricated by MPL to connect the micro-channels imprints (see Figure 6). The wafer was covered by PDMS to create the micro-fluidic device upon curing [72].

### 3.2. SU-8 Micro-Robots

Micro-robotics is a rapidly growing field focused on sub-mm sized devices that are capable of controlled locomotion, navigation, cargo delivery, and micro-manipulation. These devices are ideal for in vivo operations like targeted drug-delivery, regenerative medicine, and intercellular bio-sensing. They can be controllably actuated with propulsion mechanisms such as magnetic, electrical, chemical, ultrasound and optical stimuli [75,76]. Herein, we survey some the most recent examples of micro-robots fabricated using MPL in SU-8 that are actuated by magnetic, pH, and optical stimuli.

#### 3.2.1. Magnetic Control

Magnetic SU-8 micro-structures can be fabricated by MPL by incorporating magnetic nanoparticles (NPs) into SU-8 before MPL [77,78]. For example, superparamagnetic composites were prepared by addition of magnetite (Fe_3_O_4_) NPs to SU-8 matrix. The polymer composite was used in MPL to fabricate helical structures which could undergo corkscrew motion when a rotating magnetic field was applied. The composite was reported to be cyto-compatible with up to 10 vol.-% magnetite [77].

Another approach for magnetization is post-MPL coating with magnetic metals (e.g., Ni or Fe) [79,80,81]. A magnetically manupulated Archimedes screw was incoprtated in a micro-syringe transporter device to enable controlled collection, retention, and release of biological agents. A sacrifical cover was used to deposit Ni and Ti on the rotating screw, for magnetic actuation and cyto-compatibility, respectively, while leaving the container unmagnetized. By controllably applying a magnetic field, liquid could be forced in and out of the capsule, loading and unloading biological cargo efficiently [79].

**Figure 7:**
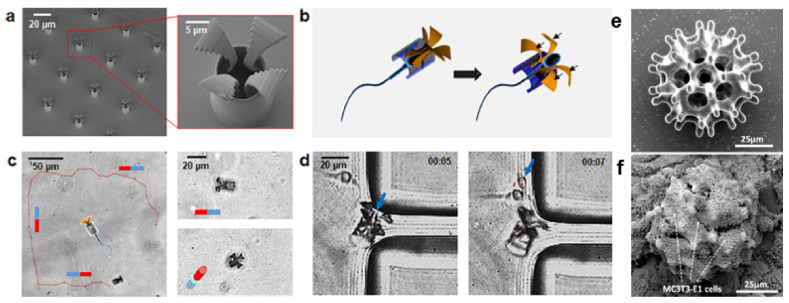
(**a**–**d**) Tetrapods fabricated by MPL. (**a**) SEM image of tetrapods. (**b**) Schematic showing the mechanical release of cell-seeded sperms upon hitting the target. (**c**) Magnetic steering of the sperm-loaded micro-robot. (**d**) Sperm release upon hitting the corner of a PDMS wall. Reproduced from Ref. [80] with the permission of ACS publications. Further permissions related to this excerpt should be directed to the ACS. Copyright 2018 American Chemical Society. (**e**,**f**) SEM image of burr-like micro-robot (**e**) as fabricated and (**f**) cultured with MC3T3-E1 cells. Adapted from Ref. [81] with permission from AAAS.

A hybrid micro-robot was made by coupling drug-carrying spermatozoa to a magenetically drivable tetropod (Figure 7a–d). The tetrapods were fabricated by MPL in SU-8 and coated with Fe and Ti for magnetization and cytocompatability (Figure 7a). Then, drug-loaded sperm were coupled into the tetrapods by swimming through them (Figure 7b). The resulting hybrid was propelled by the sperm but could be magnetically directed towards a PDMS wall (Figure 7c,d) or a cancer cell where the drug-carrying sperm was released. Using the hybrid micro-robot, the authors reported controllably released an anticancer drug into a tumor spheroid cultured in vitro [80].

Li et al. reported MPL in SU-8 burr-like porous micro-robots. It was reported that the burr-like spherical scaffold (Figure 7e) show superior cell-holding and magnetic driving capability compared to a cubic lattice. The grid length of the spherical burr was varied to maximize cell growth. After fabrication of SU-8 micro-robots, a Ni/Ti bilayer was sputter-coated for biocompatibility and magnetization. The investigators reported that these micro-robots were efficiently seeded with MC3T3-E1 cells (Figure 7f) and controllably deliver cells onto targeted areas in vivo [81].

#### 3.2.2. Controlling pH

**Figure 8:**
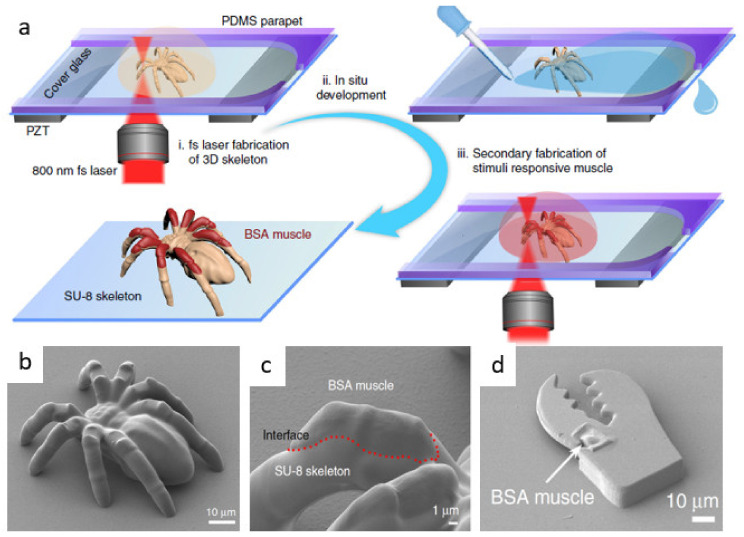
SU-8/BSA micro-robots fabricated by MPL. (**a**) Multi-material MPL scheme where the SU-8 skeleton is fabricated and developed in situ using a PDMS parapet, and fabrication is resumed after the addition of BSA. (**b**) SEM image of dynamic spider fabricated by MPL. (**c**) Magnified image showing the SU-8/BSA interface. (**d**) SEM image a claw micro-gripper actuated with pH stimulus. Figure reproduced with the permission of the authors from Ref. [88] licensed under CC BY 4.0.

Polymeric structures absorb and swell when immersed in solvents. For example, acrylic polymers swell when immersed in polar solvents like alcohols and much less in water. Although polymerized SU-8 can swell in water due to the hydroxyl groups formed upon polymerization, the dimensional variation is typically minimal and saturates in minutes [82,83]. This makes SU-8 an ideal material for microfluidic application where aqueous solutions are typically used. Additionally, SU-8 is resistant to pH variation in aqueous solutions which enables its use as a rigid pH-insensitive platform in combination with pH-sensitive materials to fabricate smart multi-component micro-devices. Bovine serum albumin (BSA) is a commercially available protein commonly used in fabrication of micro-biostructures by MPL [84,85,86,87]. The amino and carboxyl groups within the amino acid building blocks of BSA can gain or lose protons, respectively, depending on the pH. Upon (de)protonation of BSA in basic or acidic solution, the similarly charged BSA chains repel each other and cause swelling in fabricated structures. Recently, Ma et al. leveraged the controllable swelling of BSA to create dynamic musculoskeletal systems (Figure 8b) and smart micro-grippers (Figure 8d). The authors developed a novel method to fabricate multi-material integrated 3D structures by MPL. An SU-8 base structure was fabricated on the substrate and developed in situ using a PDMS parapet (Figure 8a). Fabrication software was paused prior to development and continued after insertion of BSA. This way, integrated BSA muscle could be fabricated on a rigid SU-8 base. The devices were composed of the relatively stiff SU-8 and the soft pH-sensitive BSA muscle. The investigators reported that by changing the step length in fabrication of both the SU-8 skeleton and BSA muscle, the dynamic response of the dynamic spider and the folding angle of micro-gripper could be controllably varied [88]. These smart devices were realized by engineering the swelling degree of BSA on a non-swelling rigid SU-8 platform and are promising candidates for nano-scale biological applications like targeted drug-delivery.

The same group also leveraged the BSA/SU-8 system to fabricate an artificial compound eye with tunable field of view (FOV) and focal length. Large FOV of a compound eye was augmented by addition of the vari-focal capability of the human eye. Shrinking and swelling of fabricated BSA ommatidia on top of a chemically inert SU-8 micro-lens enabled tuning of focal length from 362 μm to 242 μm without comprising FOV and the acceptance angle. The authors reported highly reversible focal length manipulation solely through varying pH. FOV was reportedly tuned from 35° to 80° and at a fixed FOV and focal length could be increased by 400% [89]. SU-8 can also be used as a template to mass produce PDMS compound eye structures [90].

#### 3.2.3. Optical Control

Optical trapping is another approach to controllably move micron and sub-micron objects [91]. Manipulating proteins and biological agents, however, is more challenging because the focused beam can damage cells. Badri et al. fabricated a four-spheroid SU-8 micro-manipulator that could be chemically attached to protein through a diamine layer. The authors reported that optical tweezing of the spheroids distanced from the attached site enabled indirect manipulation of the cell protein without damaging it [92].

### 3.3. SU-8 Optical Devices

#### 3.3.1. Micro-Lens Arrays

One approach to parallelizing MPL is to use multi-lens arrays (MLAs). Commercial MLAs have been used to create parallel focal spots at once and fabricate 2D [93] and 3D [94] patterns in reduced time. Conversely, DLW has been used to fabricate MLAs using acrylic resins [17,95]. Recently, Tsutsumi et al. used MPL to create an array of micro-lenses in SU-8, then used the fabricated MLA to write SU-8 woodpile structures in parallel. Due to very low numerical aperture of the fabricated lenses (0.2–0.3), lines within the woodpile were not resolved. However, this work showed an interesting example of the potential for insourced parallelization of MPL. SU-8 was also used for fabrication of Fresnel zone plate arrays [96] by MPL which also offer potentials for such parallelization.

#### 3.3.2. Micro-Resonators

Optical micro-resonators (OMRs) are micro-cavities than can confine resonant light to small volumes. These are typically closed-looped waveguides coupled to an optical input of another waveguide. OMRs have numerous applications including in low-threshold micro-lasers, bio-sensors, and quantum photonic devices [97,98,99,100,101]. The lifetime of the resonant photon is related to the *Q*-factor, a figure-of-merit for resonators that specifies the ratio of energy stored in the cavity to the dissipated power. The *Q*-factor of OMRs is mainly limited by surface-scattering due to the non-zero surface roughness of the cavity. A *Q*-factor as high as 10^8^ was achieved smoothing the surfaces of the cavity using selective reflow treatment with a CO_2_ laser [102].

MPL in SU-8 has been used to create OMRs, either as the sole fabrication process or part of a multi-step process. Notably, MPL was used to fabricate SU-8 masters for toroidal resonators. Using nano-imprint lithography (NIL) in PDMS, molds with negative geometry of the resonators were prepared. SU-8 or fast sol-gel materials (for silica OMRs) could then be molded into PDMS negative pattern to reproduce the original resonator geometry. Brenner et al. reported that silica resonators fabricated this way exhibit relatively high *Q*-factor (10^5^ at 635 nm) [103], comparable to that of resonators in thermoset polymer [104]. A dual-step NIL method was also proposed by the same group. Here, the master is fabricated in cross-linked SU-8 and molded into PDMS. The PDMS mold is used to replicate the master in non-crosslinked SU-8 and thermally reflowed to smoothen its surface and eliminate defects. The smooth replica was used in a second NIL using a fast sol-gel resin to create UV-cured ultra-smooth high *Q*-factor micro-resonators in silica (*Q*-factor of 3 × 10^6^), which set a record for UV-curable on-chip resonators [105]. The advantage of such multi-step processes, apart from MPL cost reduction due to NIL replication, is that sensitive materials that cannot survive MPL can be incorporated into SU-8 in the final step and patterned into a desired shape. For example, 0.5 wt.-% of the laser dye PM 567 was mixed with the sol-gel resin to create a low-threshold lasing micro-cavity [103].

#### 3.3.3. Fiber-Bound SU-8 Optical Devices

**Figure 9:**
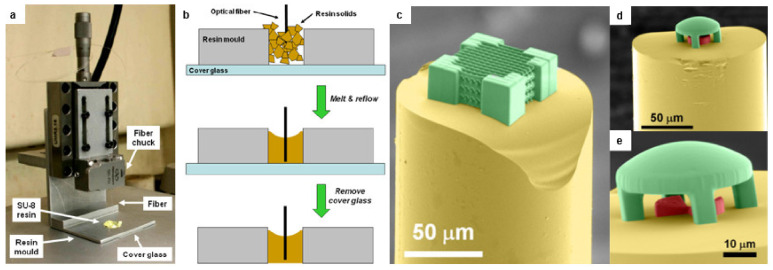
Fiber-bound SU-8 photonic devices: (**a**) Optical fiber and SU-8 resin sample mount setup; (**b**) Schematic illustration of the melt-reflow process to create SU-8 mount; False-color SEM images of (**c**) a woodpile face-centered tetragonal photonic crystal and (**d**,**e**) a compound micro-optic system consisting of a suspended plano-convex lens and a smaller plano-concave lens in contact with the fiber end-face, both fabricated on the end of an optical fiber by MPL in SU-8 resin. Reprinted with permission from [50] © The Optical Society.

Directly fabricating optical elements on top of optical fibers can greatly enhance prospects of integrated photonics. Solid photoresists like SU-8 offer advantages in handling and fabrication but are difficult to pattern on top of optical fibers because they require spin-coating and pre-baking. Williams et al. used a melt-reflow method that fabricate structures in SU-8 directly on top of optical fibers. The process involved heating SU-8 *in vacuo* to remove the volatile solvent. As shown in Figure 9a,b, the residual solids were then broken into small pieces and melted and re-flowed into an aluminum mold where an optical fiber was controllably positioned at desire length using a single-axis nano-positioner. The SU-8 in the mount bearing the fiber was then cooled to re-solidify, and the whole mount was used to perform MPL. Proof-of-concept micro-optics were fabricated on top of a fiber including convex, concave, and cylindrical micro-lenses, a compound micro-lens system, and a woodpile nanostructure (Figure 9c–e). The focusing ability of the micro-lenses was demonstrated by coupling light into the fiber [50].

#### 3.3.4. SU-8 Spatially Variant Photonic Crystals

**Figure 10:**
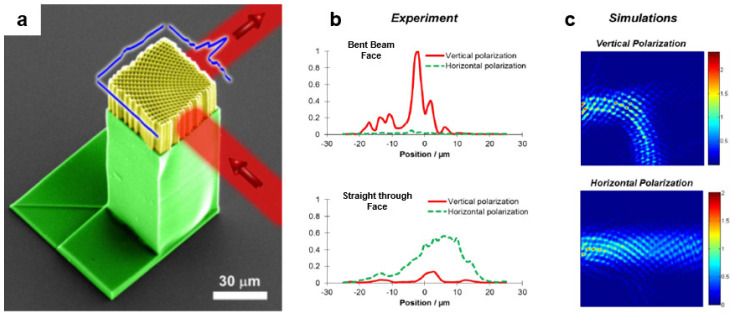
Spatially variant photonic crystals (SVPCs) fabricated by MPL in SU-8. (**a**) False-color SEM image of the SVPC (shown in yellow). The blue ribbons depict experimentally measured relative intensity exiting each face of the SVPC when an optic fiber introduced light having *λ*_0_ = 2.94 μm onto the lattice. (**b**) Polarization dependence of beam bending-for the same SVPC. (**b**) Experimentally measured intensity for vertically or horizontally polarized light exiting at faces corresponding to straight-through transmission and beam-bending. (**c**) Simulations of beam bending in the SVPC. Reprinted with permission from [58] © The Optical Society.

MPL in SU-8 has been used extensively to create photonic crystals (PCs) with stopgaps in various spectral locations in the NIR region [62,106,107,108]. MPL in SU-8 was also used to fabricate spatially-variant photonic crystals (SVPC) capable of steering light through a 90° bend [58]. The bending of light was achieved by varying the orientation of the unit cells as a function of position while maintaining lattice spacing and fill factor. Unlike reflection-based waveguides, SVPCs can bend light with a radius of curvature as low as 6.4*λ* where *λ* is the vacuum wavelength of light (Figure 10a). Further, there are no limitations on the size or the profile of the beam. Diffraction-based SVPCs maintain the shape and the composition of the unit cell and the fill factor, thereby reducing the fabrication cost compared to graded photonic crystal and graded-index materials [58]. Additionally, SVPCs allowed control of polarization of the output beam as the vertically polarized light was bent effectively, while horizontally polarized light virtually passed straight through (see Figure 10b). The polarization sensitivity behavior was predicted by electromagnetic simulations (Figure 10c) [109] and could be turned off by using a symmetric unit cell [58].

## 4. Metallization of SU-8 Micro-Structures

### 4.1. Local Metallization using Double Resist Layer

**Figure 11:**
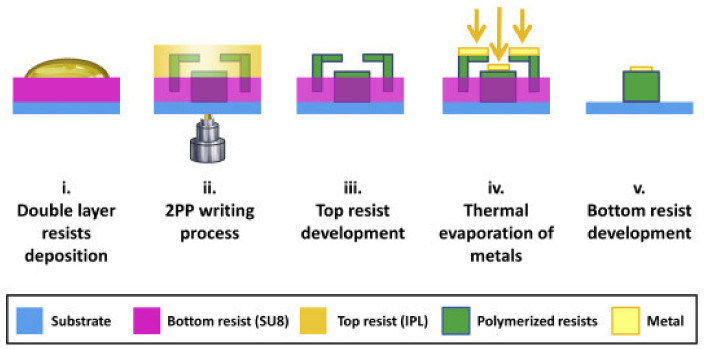
Schematic illustration of localized metallization using a sacrificial stencil mask. Reproduced with permission from Ref. [110] Copyright 2019 Elsevier.

Puce et al. recently reported a process based on simultaneous fabrication of a polymeric scaffold and a sacrificial stencil mask that can be used for local deposition of a metal. The process is shown in Figure 11. Two layers of photoresist were cast on a glass substrate. The scaffold and the sacrificial mask were then written into both layers by MPL. Polymerized IP-L was developed in IPA which left the SU-8 layer (both polymerized and un-polymerized) intact. After local deposition using the sacrificial mask, the mask could then be lifted off to yield a locally coated polymeric scaffold. While the mask can be fabricated ex situ and used for local deposition, this integrated approach eliminates the laborious alignment of the mask with the fabricated scaffold. Interestingly, the authors noticed that the IP-L resin diffuses significantly into the prebaked SU-8 layer prior to MPL process. To remedy this issue, a diffusion barrier was created by UV-exposing the SU-8 for 8 s prior to adding IP-L. However, the polymerized “skin” on the SU-8 layer reportedly made it more difficult to remove the mask and necessitated use of ultra-sonication [110].

### 4.2. SU-8 Functionalized with Gold Nanoparticles

**Figure 12:**
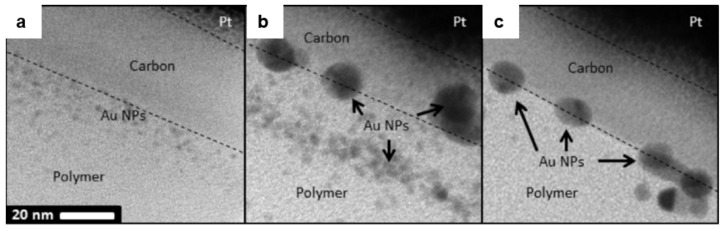
TEM bright-field cross-sectional images of Au-NP-functionalized SU-8 films prepared using the reducing agents (**a**) NaBH_4_, (**b**) sodium citrate, and (**c**) hydroquinone. Reprinted with permission from Ref. [114] Copyright 2014 Elsevier.

Gold nanoparticles (Au NPs) are useful for signal enhancement via surface plasmon resonance with applications in bio-sensing and cell imaging [111,112,113]. Clukay et al. demonstrated on- and sub-surface functionalization of SU-8 with Au NPs [114]. SU-8 epoxide groups were immersed in multi-functional amines to create pendant amines on the surface. The aminated surface was immersed in a chloroauric acid bath whereupon the pendant amines coordinated to Au ions. The Au ions bridged to the surface were subsequently reduced in situ with control over their size and distance from the interface at which they formed. The authors reported that the strength and mobility of the reducing agent, as well as the size of the amine can affect the depth and size of deposited Au NPs [114]. The size of Au NPs could be controlled by judicious choice of the reducing agent (Figure 12). NaBH_4_ (a strong reducing) agent formed small NPs (2–5 nm) and hydroquinone (a mild reducing agent) formed larger NPs (5–10 nm) [115]. Intermediate behavior was observed when sodium citrate was used as the reducing agent. This behavior was explained by the fact that the growth of Au NPs inside the SU-8 matrix is diffusion-limited while their nucleation is not. Additionally, the depth of Au-NPs from the polymer interface correlated with the size (and hence mobility) of the reducing agent. Further, the depth of Au NPs layer could be controlled by varying the size of the amines when the same reducing agent was used. Ethylenediamine diffused more easily and formed dispersed Au NPs up to 20 nm deep while larger amines (e.g., tetraethylenepentamine, EPA) diffused less and form a single layer of Au NPs at the polymer interface [114].

### 4.3. Improved Amination Process for Less Distorted Primed SU-8

**Figure 13:**
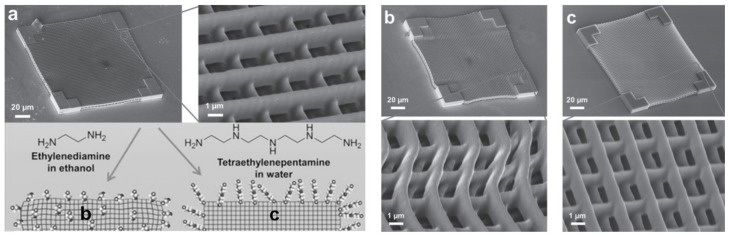
SEM images of SU-8 micro-lattices (**a**) as-fabricated, (**b**) after conventional amination with ethylenediamine in ethanol and (**c**) after amination using TEPA in water. Figure adapted with permission from Ref. [83] Copyright 2014 John Wiley and Sons.

Amination is a common step to prime the epoxide surface of SU-8, so that it can be further functionalized, including metallization. Amination can be carried out by simply immersing the SU-8 surface in a solution of an alkyl diamine (e.g., ethylenediamine) in ethanol. Kuebler et al. reported that this procedure causes irreversible shrinkage and distortion in SU-8 micro-lattices fabricated by MPL because as the ethanol solution of ethylenediamine infuses and swells the structure, it can react with unpolymerized oligomers and wash them away, causing irreversible distortion (Figure 13b) [83]. To remedy this problem, the authors investigated aqueous amination solutions which resulted in much lower irreversible distortion upon amination (Figure 13c) [83]. Water is not a good solvent for SU-8 and similar polymers compared to alcohols and therefore does not swell the SU-8 polymer as much [82]. Additionally, the authors reported that using higher-molecular-weight alternatives to ethylenediamine could hinder diffusion of the amine into the cross-linked polymer and limit amination to the SU-8 surface. SU-8 structures aminated using tetraethylenepentamine (TEPA) in aqueous solution (Figure 13c) showed no further distortion compared to the as-fabricated sample (Figure 13a) without comprising the surface density of amine group used for metallization [83].

### 4.4. Silver Functionalized SU-8 Micro-Structures

**Figure 14:**
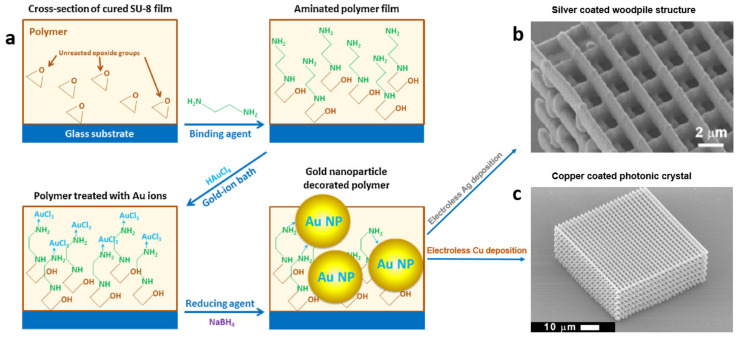
Au-NP-assisted conformal deposition of metals on the surface of SU-8 micro-structures fabricated by MPL. (**a**) Schematic illustration of Au NP deposition on SU-8 surface. Reprinted with permission from Ref. [114] Copyright 2014 Elsevier. (**b**) SU-8 woodpile structure fabricated by MPL and coated with silver via Au-NP-assisted electroless deposition. Reprinted with permission from Ref. [116] Copyright 2007 American Chemical Society. (**c**) SU-8 photonic crystal fabricated by MPL and metallized with copper via Au-NP-assisted electroless deposition. Reprinted with permission from Ref. [34] © The Optical Society.

Importantly, Au NPs can also be used as nucleation sites for deposition of other metals. Chen et al. demonstrated silver coating of polymeric woodpile structure via electroless deposition of silver on Au-NP-functionalized SU-8. Microstructures in SU-8 fabricated by MPL can be functionalized with Au NPs per the aforementioned procedure and immersed in a buffered bath of silver lactate, hydroquinone, and gum arabic for silver deposition (Figure 14) [116]. This process is also applicable to methacrylate and acrylate surfaces by using a lithium diamine (i.e., the aminolysis product of an alkyl lithium and a diamine) to create the primed SU-8 surface for Au-NP formation [117]. Acrylate surface can also be aminated (and therefore primed for Au- functionalization) using Michael addition reaction. This way, a complex device fabricated on a single substrate using both methacrylic and acrylic resins can be selectively functionalized [118] because methacrylates do not undergo Michael addition. The morphology and the size of silver particles on the coating can be engineered by manipulating the bath composition. Grabill et al. explored the effect of Ag concentration, the nature of carboxylate species used to buffer the electrodeposition, and the presence of gum arabic (a glycoprotein traditionally used to increase solubility of silver citrate) on the size of silver NPs and the morphology of their agglomerates, the rate of deposition, and the stability of the bath. Further, a highly stable maleate bath formulation stable for several hours was reported [119].

### 4.5. Copper-SU-8 Photonic Crystals

Conformal Cu coating on the SU-8 structures was also demonstrated by electroless deposition on the primed (Au-NPs-functionalized) polymeric surfaces (see Figure 14c). This capability enabled facile fabrication of metallo-dielectric photonic crystals (PCs) exhibiting a photonic band gap (due to periodicity of the PC), as well as a plasmonic band-edge (due to Cu coating) [34,120]. Ni-coated SU-8 PCs were also fabricated by MPL in SU-8 followed by electroless deposition [121].

## 5. Novel Techniques for Enhancing Resolution of SU-8

By carefully controlling incident laser power, Juodkazis et al. [122] showed it was possible to create fine suspended fibers by MPL in SU-8, some of which had feature-width as small as 30 nm. Similarly small features have been reported for MPL in acrylate resins using visible pulsed beams (<65 nm) [123], ultrashort sub-10 fs pulses (<50 nm) [124], and ultrafast scanning (<25 nm) [125]. In these cases, the reported minimum feature size represents the smallest feature that was observed and not the smallest that could be reproducibly fabricated, nor the smallest feature that was mechanically robust and self-supporting. Resolution is more commonly defined as the minimum distance that can be achieved between two separately and reproducibly written features. Importantly, this measure differs from and is greater than the minimum width achievable for isolated features. As the focal spot of the laser scans within the resin, active species are generated in neighboring non-polymerized regions, where the concentration of initiating species generated does not reach the threshold for solidification. These species lower the polymerization threshold near previously written features, and consequently also lower the resolution that can be achieved for non-isolated features. This is sometimes referred to as the *memory effect* [126]. Adjacent exposure also lowers the damage threshold of the material, which some refer to as the *proximity effect* [127]. To address the memory effect and improve the resolution of MPL, various schemes have been investigated in acrylate systems. Inspired by stimulated emission depletion (STED) microscopy, Fourkas et al. used a single color beam for both activation and deactivation and feature size of 40 nm was achieved [128]. Wegener et al. has also employed a second doughnut-shaped beam (of a green CW laser) to peripherally de-excite the photoinitiators activated by the first beam (red pulsed) and thereby improve the resolution [129,130,131]. Another approach to improve resolution has been diffusion-assisted quenching [132] which leverages a mobile inhibitor used with a low-diffusing monomer system to reduce the memory effect by inhibitor diffusion when low writing speeds are used.

### 5.1. Self-Quenching SU-8 PAG

**Figure 15:**
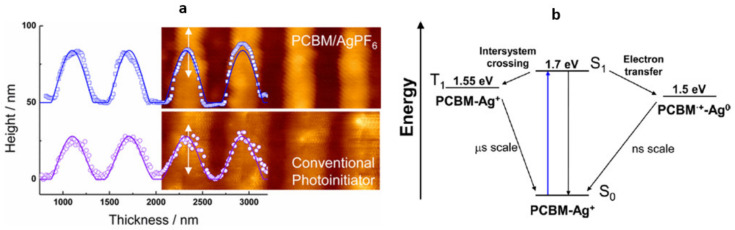
(**a**) overlapped AFM images and height profiles of lines fabricated by MPL in SU-8 using PCBM/AgPF_6_ system and conventional PAG. (**b**) Energy diagram and proposed decay pathway of PCBM/AgPF_6_ system upon excitation. Reprinted with permission from Ref. [133] Copyright 2017 American Chemical Society.

Recently, a novel scheme for improving resolution based on reversible cationic photoinitiation was proposed to improve resolution of SU-8 lines in MPL [133]. Instead of a PAG, which irreversibly forms Brønsted acid upon excitation, a C60-based PI, [6,6]-phenyl-C61-butyric acid methyl ester (PCBM), was used with oxidizing agent AgPF6. Duocastella et al. reported that cationic species formed by electron transfer from the excited state occurring in nanoseconds. Residual cations relax by intersystem crossing and revert back to the ground state within the μs timescale, so the polymerization threshold for a neighboring line is not affected. The resolution of lines fabricated in SU-8 has been improved compared to PAG-initiated control from 600 nm down to 400 nm (Figure 15a). The achieved resolution is comparable to that of diffusion-assisted quenching approach [132] and offers a new pathway for improving resolution in single-beam MPL. The exact cationic mechanism is not yet known and requires further exploration.

### 5.2. In Situ Post Exposure Bake within the Polymerized Features

SU-8 differs from acrylic-based MPL resins in that it requires post-exposure baking (PEB) to activate cationic polymerization of micro-structures. The fact that exposed SU-8 requires heating to polymerize creates an additional step that lengthens the fabrication time, but it can be used as an advantage. The same laser light that excites PAGs can potentially locally heat a portion of irradiated region beyond PEB temperature of SU-8 (*T* > 95 °C). High intensities of light can create local elevated temperatures due to thermal accumulation. Local heating is more pronounced for continuous wave (CW) lasers and pulsed lasers with higher repetition rates and longer pulse durations, where the time between pulses is smaller than heat dissipation time [134].

Seet et al. demonstrated partial optical curing of MPL fabricated SU-8 lines due to local heating at high irradiances. With the elimination of PEB, the authors reported that features could be miniaturized by a factor of two compared to the conventionally post baked samples [49].

Linear absorption of SU-8 in the visible region is minimal but non-zero. Highly focused beams of CW lasers emitting at 532 nm [135,136,137] and 442 nm [138] have been employed to induce polymerization of SU-8 only in the focal region of the laser. Nguyen et al. reported in situ optical curing of SU-8, driven by 1PA-induced heating, and created woodpile structure with 400 nm periodicity [136]. This method was also used to fabricate SU-8/Fe_2_O_3_ magnetic composite pillars [78]. Thiel et al. reported a power dependence for curing SU-8 which is consistent with 2PA when fabricating features using CW laser emitting at 532 nm, whereas a mechanism by which a third-order process could take place at such lower powers (10 mW) was not discussed [137]. The order of excitation of SU-8 under different irradiation conditions and the effect of thermal heating on various non-linear phenomena requires further investigation.

### 5.3. Simultaneous Spatio-Temporal Focusing

Chu et al. applied the method of simultaneous spatiotemporal focusing (SSTF) based on spatio-temporal chirping of the femtosecond pulses [139,140] to MPL and successfully fabricated centimeter-high structures in SU-8 [141]. In typical MPL the height of the structure fabricated by MPL is inherently limited by the high-*NA* objective that must be used to achieve proper focusing. Thus, the working distance of the objective restricts the structure height to a few mms.

Another limitation of MPL is that the voxel is asymmetric, and the axial length (along the direction of beam propagation) is typically three times larger than the lateral dimensions when the beam is focused using high-*NA* optics (*NA* > 1). Isotropic features can therefore be written only by multi-line scanning. Using SSTF, Chu et al. also reported isotropic spatial resolution (spherical focal spot). Isotropic features could be linearly tuned 10–44 μm by merely varying the laser power [141].

## 6. Conclusions and Outlook

Over the last two decades SU-8 has steadily grown in importance as a material system for the fabrication of functional microdevices. This growth is supported by the compatibility of SU-8 with MPL and the capabilities they offer together for creating complex, robust 3D structures. Working with SU-8 has enabled facile integration of MPL with other lithographic methods, which has particularly helped multi-scale fabrication of microfluidic devices. Advances in SU-8 post-processing, such as conformal metallization and reflow techniques, have supported its wider use for MPL of micro-robots, optics, and photonic devices. Novel formulation and processing of SU-8 have shown promise for improving the resolution attainable by MPL. There are numerous opportunities for further work in the field. Acrylate-based materials offer higher resolution for MPL. Developing new approaches for improving resolution with SU-8 would broaden its use. One such approach is to modify the composition of SU-8 for higher compatibility with MPL. Self-quenching PAGs have shown promise in lowering the resolution of SU-8, yet their optical non-linearity and activation/deactivation mechanisms are not yet fully understood. Nonetheless, it is anticipated that such smart PAGs will be a focus of future research in MPL using SU-8. The effect of the type and concentration of SU-8 solvent, and novel processing for removal of solvent before exposure is also a promising means for improving resolution. Loss of resolution has been attributed to higher diffusivity of acid when residual solvent is present in the pre-exposed material. Novel drying techniques and other approaches could be used to suppress acid diffusion and improve resolution.

Substantial progress can be realized by developing new composites based on SU-8 that incorporate semiconductors, metals, active chromophores, and other functionalities that imbue structures with active function. There should be many opportunities for discovery and advancement by exploring additional ways in which SU-8 and MPL can be used to create bio-architectures, scaffolds for cell- and tissue growth, and medical devices and sensors. In the last few years, new pathways for fabrication of SU-8 based smart microdevices have emerged. By using in situ development, sequential multi-material fabrication of SU-8 with functional photoresists (e.g., with pH-sensitive proteins) has been achieved. Because cross-linked SU-8 is rigid, compatible with most polymeric surfaces, and non-swelling in aqueous media, it can be employed as an inert platform on which functional micro-parts can be incorporated. SU-8 is also optically transparent and non-magnetic, so additional approaches for creating composites and SU-8 hybrids containing magnetic species should enable fabrication of smart microsystems that can be actuated with a variety of magnetic and electromagnetic stimuli.

## Data Availability

Not applicable.
